# Molecular Detection and Identification of *Babesia* spp., *Theileria* spp., and *Anaplasma* spp. in Sheep From Border Regions, Northwestern China

**DOI:** 10.3389/fvets.2020.00630

**Published:** 2020-09-16

**Authors:** Yongchang Li, Eloiza May Galon, Qingyong Guo, Mohamed Abdo Rizk, Paul Franck Adjou Moumouni, Mingming Liu, Jixu Li, Shengwei Ji, Bayin Chahan, Xuenan Xuan

**Affiliations:** ^1^National Research Center for Protozoan Diseases, Obihiro University of Agriculture and Veterinary Medicine, Obihiro, Japan; ^2^Parasitology Laboratory, Veterinary College, Xinjiang Agricultural University, Urumqi, China; ^3^Department of Internal Medicine and Infectious Diseases, Faculty of Veterinary Medicine, Mansoura University, Mansoura, Egypt

**Keywords:** *Theileria*, *Babesia*, *Anaplasma*, sheep, China

## Abstract

*Babesia, Theileria*, and *Anaplasma* are important causative agents of tick-borne diseases that severely affect sheep. However, there is paucity in the occurrence genetic diversity of the infections of tick-borne diseases in sheep in border regions, northwestern China. In this study, nested polymerase chain reaction (nPCR) assays and gene sequencing were used to identify tick-borne *Babesia* spp., *Theileria* spp., and *Anaplasma* spp. infections in border regions, northwestern China. Out of 323 samples tested in this study, 225 (69.7%) sheep were infected with *Babesia* spp., *Theileria* spp., and *Anaplasma* spp. Two hundred six (63.8%), 60 (18.6%), 54 (16.7%), 51 (15.8%), 32 (9.9%), 19 (5.9%), and 16 (5.0%) were positive for *A. ovis, B. motasi*-like, *A. bovis, T. uilenbergi, A. phagocytophilum, T. luwenshuni*, and *B. motasi*-like Xinjiang, respectively. The most common dual infection was with *A. ovis* and *B. motasi-*like while the most frequent triple coinfection was *A. ovis, B. motasi*-like, and *T. uilenbergi* with coinfection rates of 17.0% (55/323) and 5.0% (16/323), respectively. Sequencing analysis indicated that *A. ovis MSP4, A. phagocytophilum epank1, A. bovis* 16S rRNA, *B. motasi*-like *rap1-b, B. motasi*-like Xinjiang *rap1-a, T. luwenshuni* 18S rRNA, and *T. uilenbergi* 18S rRNA from border regions, northwestern China, showed 99–100% identity with documented isolates from other countries. To the best of the authors' knowledge, this is the first report of *T. uilenbergi* and *T. luwenshuni* infections of sheep in border regions, northwestern China. Furthermore, these findings provide important data for understanding the distribution of *Babesia, Theileria*, and *Anaplasma* in sheep between border countries and China.

## Introduction

Babesiosis, theileriosis, and anaplasmosis are important diseases caused by pathogens transmitted by ticks (1). Two generally species of *Babesia* species (*Babesia motasi* and *B. ovis*) (2, 3) and several species of *Theileria* (*Theileria ovis, T. lestoquardi, T. sespecies, T. luwenshuni*, and *T. uilenbergi*) of ovine babesiosis and theileriosis are distributed in Asia, Africa, Europe, and the Far East (4–6). Furthermore, ovine anaplasmosis is mainly caused by *A. ovis* and *A. marginale* (7, 8). Clinical signs of babesiosis, theileriosis, and anaplasmosis in sheep and goats include anemia, fever, abortion, weight loss, reduce milk supply, jaundice, and even death, causing huge economic losses for sheep, and goat production (3, 5, 7).

In China, *Babesia motasi, B. ovis* (9, 10), *Theileria ovis, T. uilenbergi, T. luwenshuni* (4–6), *Anaplasma phagocytophilum, A. marginale, A. ovis, A. bovis, A. capra*, and *A. platys* (11, 12) are the causative agents of ovine tick-borne disease transmitted by ticks or small ruminant that have been reported.

Xinjiang Uygur Autonomous Region (XUAR), an area surrounded by multiple land forms including Gobi desert, valley, mountain, grassland, and plates, occupies one-sixth of China's land area and borders eight countries including Russia, Mongolia, Kazakhstan, Kyrgyzstan, Tajikistan, Afghanistan, Pakistan, and India (13). According to Song et al. (14), in 11 border counties of XUAR, five *Babesia* species, two *Theileria* species and *A. ovis* were identified in *Haemaphysalis punctata, Hyalomma asiaticum, Dermacentor nuttalli, D. marginatus, Rhipicephalus turanicus*. Furthermore, in 2010, there were more than 30 million sheep and goats in XUAR (15), but information about ovine tick-borne pathogens is still lacking in border regions, northwest China. Subsequently, this study was conducted to help in full filling the information gap regarding the occurrence and genetic diversity of *Babesia* spp., *Theileria* spp., and *Anaplasma* spp. in sheep from northern and southern XUAR.

## Materials and Methods

**Figure 1:**
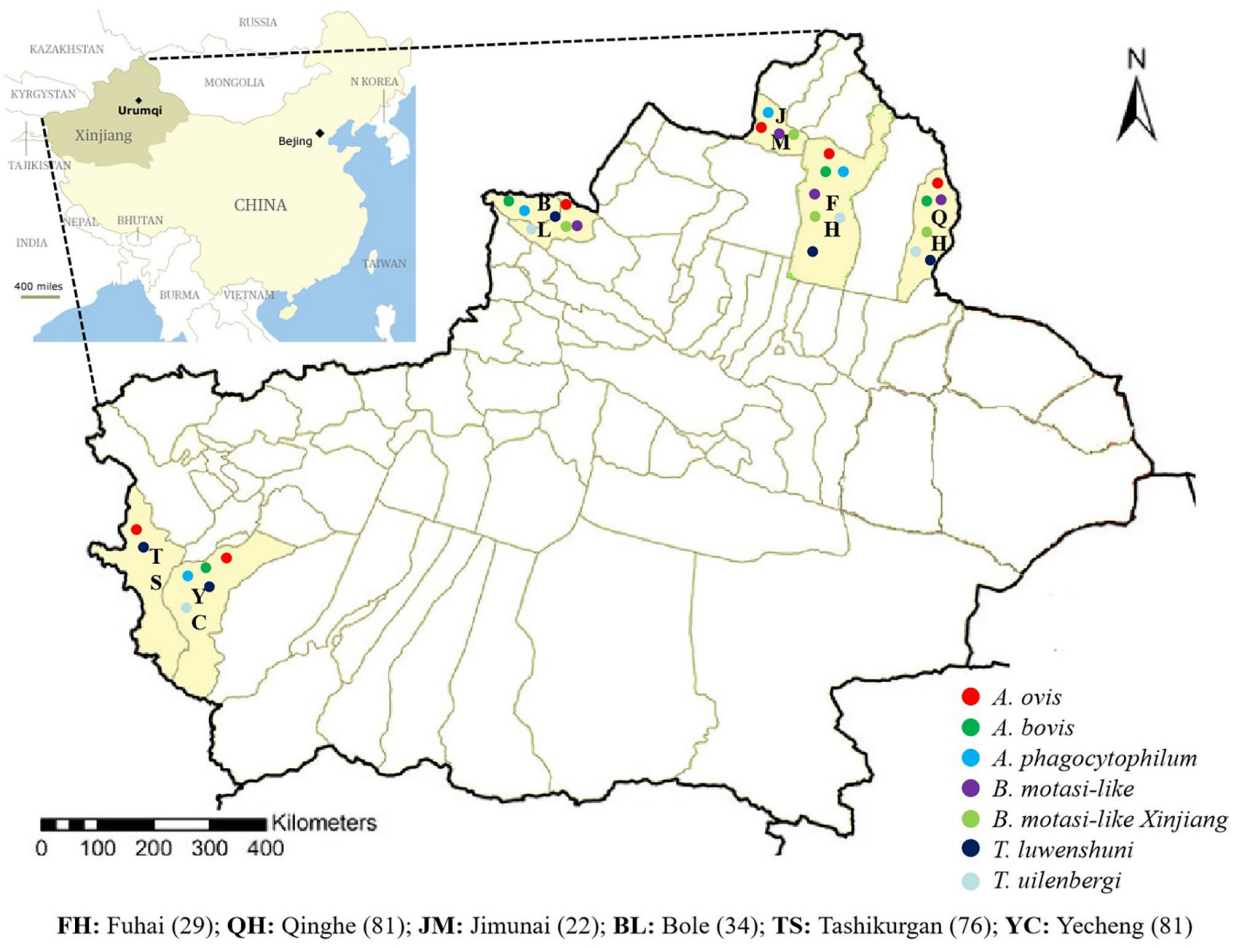
Map of XUAR, China. Dots indicate localities which pathogens were detected.

Sheep blood samples (*n* = 323) were collected from Fuhai (FH, *n* = 29), Qinghe (QH, *n* = 81), Jimunai (JM, *n* = 22), Bole (BL, *n* = 34), Tashikurgan (TS, *n* = 76), and Yecheng (YC, *n* = 81) between June 2018 and June 2019 in the southern and northern XUAR (Figure 1). The age ranges of sampled sheep were 113 (0– ≤ 2 years old), 184 (>2– <7 years old), and 26 (≥7 years old) in 292 female and 31 male sheep. After collection, blood samples were transported to the laboratory in cool boxes and kept at 4°C. DNA was extracted from 200 μl of whole blood using the QIAamp DNA Blood Mini Kit (Qiagen, Germany), following the manufacturer's protocol and stored at −30°C until used.

All samples were screened with species-specific primers for *Theileria* spp., *Babesia* spp., and *Anaplasma* spp. by PCR or nested PCR (nPCR) (Table S1). Based on fragments of *major surface protein 4* (*MSP4*) gene of *A. ovis* and *A. marginale, epank1* gene of *A. phagocytophilum*, 16S rRNA gene of *A. bovis* and *A. capra*, 18 ssu rRNA gene of *B. ovis* and *T. ovis, rhoptry-associated protein* (*rap*)*1-b* gene of *B. motasi*-like, *rap1-a* gene of *B. motasi*-like Xinjiang, 18S rRNA of *T. luwenshuni* and *T. uilenbergi*, and *major piroplasm surface protein* (*MPSP*) of *T. orientalis*. The PCR reaction mixture was composed of the following Takara super mix (Takara, Japan), PCR products were checked by electrophoresis in 1.5% agarose gels. Five positive samples were randomly selected from the six sampling areas, and at least three clones were sequenced. When the sequences obtained from different amplicons were identical, only one amplicon was retained for sequence analysis. Representative amplicons were cloned in pGEM-T Easy Vector. In brief, after extracting the amplicons from the agarose using QIAquick Gel Extraction Kit (Qiagen, Germany), pGEM-T Easy Vector (Promega, USA), and NucleoSpin® Plasmid QuickPure (Macherey-Nagel, Germany). BigDye Terminator Cycle Sequencing Kit (Applied Biosystems, USA) and 3100 Genetic Analyzer (Applied Biosystems, USA) were used for sequencing (13). The nucleotide sequence identities were determined by performing GenBank BLASTn analysis on the NCBI database. The confidence interval (95% CI) analyses were conducted using GraphPad Prismer 6.0.

## Results and Discussion

Among the 323 samples, the PCR assays revealed that 225 (69.7%) sheep were infected with *Babesia* spp., *Theileria* spp., and *Anaplasma* spp. The overall infection rates of sheep are presented in Table 1. The most frequent pathogens were *A. ovis* detected in 63.8% of the samples, followed by *B. motasi*-like (18.6%), *A. bovis* (16.7%), *T. uilenbergi* (15.8%), *A. phagocytophilum* (9.9%), *T. luwenshuni* (5.9%), and *B. motasi*-like Xinjiang (5.0%) (Table 1). The most common coinfection was *A. ovis* and *B. motasi*-like with an infection rate of 17.0% (55/323), followed by *A. ovis* and *T. uilenbergi* with 12.4% (40/323). Meanwhile, only one sheep was dual infected with *A. phagocytophilum* and *T. luwenshuni*. For triple coinfection, the infection rate of *A. ovis* and *B. motasi*-like and *T. uilenbergi* was 5.0% (16/323) (Table S2).

**Table 1:** Detection of *Anaplasma* spp., *Babesia* spp., and *Theileria* spp. in sheep from XUAR, China.

**County**	**No. tested**	**No. Infected/% (95% CI)**						
		***A. ovis***	***A. bovis***	***A. phago***	***B. m-*like**	***B.m* Xinjiang**	***T. luwenshuni***	***T. uilenbergi***
TS	76	1/1.3 (−0.01–0.04)	–	–	–	–	1/1.3 (−0.01–0.04)	–
BL	34	32/94.1 (0.86–1.03)	8/2a3.5 (0.18–0.38)	7/20.6 (0.06–0.35)	26/76.5 (0.61–0.91)	12/35.3 (0.21–0.50)	3/8.8 (−0.01–0.19)	17/50.0 (0.32–0.68)
FH	29	26/89.7 (0.78–1.01)	11/37.9 (0.22–0.52)	1/3.4 (−0.04–0.11)	9/31.0 (0.13–0.49)	3/10.3 (0.03–0.17)	7/24.1 (0.09–0.38)	11/37.9 (0.19–0.56)
JM	22	18/81.8 (0.64–0.99)	–	1/4.5 (−0.05–0.14)	4/18.2 (0.01–0.36)	1/4.5 (−0.05–0.14)	–	–
YC	81	64/79.0 (0.70–0.88)	20/24.7 (0.16–0.34)	23/28.4 (0.18–0.38)	–	–	3/3.7 (−0.03–0.10)	10/12.4 (0.05–0.20)
QH	81	65/80.2 ()	15/18.5 (0.10–0.27)	–	21/25.9 (0.16–0.36)	10/12.3 (0.05–0.20)	5/6.2 (0.01–0.12)	14/17.3 (0.08–0.26)
Total	323	206/63.8 (0.59–0.69)	54/16.7 (0.13–0.21)	32/9.9 (0.07–0.13)	60/18.6 (0.14–0.23)	16/5.0 (0.03–0.07)	19/5.9 (0.03–0.08)	51/15.8 (0.12–0.20)

“*–” mean not detected; TS, Tashikurgan; Bl, Bole; FH, Fuhai; JM, Jimunai; YC, Yecheng; and QH, Qinghe*.

All *A. ovis, A. bovis, A. phagocytophilum, B. motasi*-like, *B. motasi*-like Xinjiang, *T. luwenshuni*, and *T. uilenbergi* sequences in this study were the expected sizes of 347, 551, 444, 536, 507, 389, and 388 bp, respectively. The *MSP4* gene sequences of *A. ovis* (GenBank accession numbers MN946542) were 97.9% identical to other and to an isolate from Sudan (KU497712). *A. bovis* 16S rRNA gene sequences (GenBank Accession no. MN947620) was 99% identical to an isolate obtained from cattle in China (MK345480). *A. phagocytophilum epank1* gene sequences (GenBank Accession no. MN946539) were 98% identical to an isolate obtained from sheep in Germany (GU236795). Sequences of *B. motasi*-like *rap1-b* sequences (GenBank Accession nos. MN946540) showed 99% identity to a previous isolate from XUAR sheep (KU510048). The sequence identity of *B. motasi*-like Xinjiang *rap1-a* (GenBank Accession no. MN946541) with an isolate from *Haemaphysalis longicornis* in Gansu, China (KX708614), was 99%. The accession numbers for 18S rRNA of *T. luwenshuni* and *T. uilenbergi* are MN944535 and MN944557, respectively. The complete list of GenBank accession numbers of sequences from this study is shown in Table S3.

Tick-borne pathogens (TBPs) of ovines represent a serious threat to veterinary and public health worldwide (16–18). In this study, three species of *Anaplasma* and two species of *Babesia* and *Theileria* were molecularly detected from sheep. The result also revealed that 69.7% of the samples were infected with at least one pathogen. Generally, the relatively high incidence of TBPs could be attributed to the abundance of vectors in the study area. In XUAR, 45 species from 6 genera of ticks, namely, *Hyalomma, Dermacentor, Haemaphysalis*, and *Rhipicephalus* are reported (19). In addition, *Rhipicephalus turanicus, Dermacentor niveus, Hyalomma asiaticum*, and *Dermacentor marginatus* are the most frequent tick species in domestic animals from thirty-five counties (cities) in XUAR during 2011–2017 (20).

A number of *Anaplasma* species including *A. ovis, A. bovis*, and *A. phagocytophilum*, have been documented to cause anaplasmosis in ruminants (21). In this study, we identified *A. ovis* (63.8%), *A. bovis* (16.7%), and *A. phagocytophilum* (9.9%). Previously, (11) found *A. ovis* and *A. phagocytophilum* in sheep in XUAR, while *A. ovis* was identified in ticks in border regions (14). In addition, *A. ovis* and *A. bovis* were identified in Fuhai and Qinghe while *A. phagocytophilum* was detected in Fuhai, which both border Mongolia. *A. ovis* was identified in sheep and *Dermacentor nuttalli* tick (22) and *A. phagocytophilum* in *I. persulcatus* in areas surrounding Mongolia (23). We also detected *A. ovis, A. bovis*, and *A. phagocytophilum* in Bole and *A. ovis* and *A. phagocytophilum* in Jimunai which both areas are adjoined to Kazakhstan. According to Shpynov et al. (24), *A. phagocytophilum* DNA was identified in *I. persulcatus* ticks in the Altai and Primorye territories, which is also borders Kazakhstan. Furthermore, *A. ovis* (79.0%), *A. bovis* (24.7%), and *A. phagocytophilum* (28.4%) in Yecheng sheep in current study, while *A. ovis* (1.5%), *A. marginale* (5.7%), and *A. centrale* (2.7%) were identified in ticks from Pakistan, the country that Yecheng borders (25). Other border countries of XUAR also have reports about *Anaplasma* infection (*A. bovis, A. centrale, A. phagocytophilum*, and *A. marginale*) in ruminants or ticks including Russia (26), Philippines (27), and south India (28). Notably, *A. capra*, a newly discovered emerging zoonotic species of *Anaplasma*, was not identified in the current study, although it has been reported in both XUAR (only four positive samples) and Gansu of China (29, 30). Such discrepant is attributed to the difference in the area where the samples were collected in our study and in the previous one in XUAR (29).

Several *Babesia* species (*B. motasi*-like and *B. ovis*) are widely spread among sheep and goat in China, specifically in Gansu and Qinghai (31, 32), consistent with the distribution of the tick vectors (33). The total infection rates were 0.4% for *B. motasi* in sheep from Gansu according to (34). Song et al. (14) reported that in XUAR, five *Babesia* were identified in *H. asiaticum, H. punctata, D. nuttalli*, and *R. turanicus* ticks which were collected from sheep, including *B. occultans, B. motasi*-like, and *B. major*. Meanwhile, none of the samples was positive for *B. motasi*-like in ticks which were collected from Fuhai, Jimunai, Qinghe, and Yecheng (14). However, except in Yecheng, we found *B. motasi*-like infection in sheep in Fuhai (31.0%), Jimunai (18.2), and Qinghe (25.9%). According to Niu et al. (31), the recorded infection rate for *B. motasi*-like was 21.87%, while Song et al. (14) also reveal that *B. motasi*-like was identified in *H. punctata* ticks in Yili district. In countries nearby XUAR, such as Pakistan, *B. ovis* (16%) and *T. ovis* (23%) were also detected from sheep (35).

The first infection by *Theileria* spp. in small ruminants imported into Sichuan province of China was reported in 1958 (36). Infection with other *Theileria* species, such as *T. uilenbergi, T. luwenshuni*, and *T. ovis*, infective to small ruminants, has been documented since the first report (34). Furthermore, *T. luwenshuni* and *T. uilenbergi* were found in Gansu and Sichuan, Heilongjiang, Qinghai, Hubei, and Hainan, China (12, 16, 32). In this study, pathogenic *T. luwenshuni* was detected in sheep from *five* counties (Fuhai, Bole, Qinghe, Yecheng, Tashikurgan), with detection rates of 24.1, 8.8, 6.2, 3.7, and 1.3%, respectively. Although *T. ovis* (7.25%) was previously detected in *R. turanicus* ticks infesting sheep in Yecheng (14), we only identified *T. luwenshuni* and *T. uilenbergi* in sheep from Yecheng in this study. *T. lestoquardi* was also found infecting apparently healthy sheep and goats from two districts of Pakistan, which was near Yecheng county (37). In addition, *T. luwenshuni* infection in ticks from dogs and cattle was identified by reverse line blot hybridization (34). Aktaş et al. (38) revealed the presence of *T. orientalis* and *T. annulata* from cattle in Kyrgyzstan, which is near Bole. In south India, which is near Yecheng and Tashikurgan, researchers also identified *T. orientalis* in ticks (29). In previous studies, *T. ovis* were found in ticks (39) and goats (40) from XUAR, but in the present study, all sheep DNA samples showed a negative result for *T. ovis* and *T. orientalis*. The reasonable explanation for such obtained finding might be attributed to the difference between collection sample areas and tick species with previous study in XUAR.

The distribution and diversity of pathogens in sheep may be attributed to the difference in macroclimate, tick species, tick-dwelling habitat, and landscape between districts. Particularly, questing ticks can be found in habitats like herb layer and vegetation (41, 42). In XUAR, cattle, horses, and sheep often co-feed in the same district, and there are also many small mammals in areas with high vegetation (43). These expand the possibility of ticks finding an animal as its host, but reduce the possibility of ticks encountering the same animal (44). Furthermore, the districts containing alpine and meadow climates have humid soils rich in organic matter resulting in abundant vegetation which favors the tick population (42, 45). Those aspects might explain why a lot of TBPs and different tick species were identified in XUAR. Although this study screened several TBPs in sheep reared in the southern and northern parts of XUAR, China, this survey did not include ticks from this area. Therefore, other future studies are required to detect those pathogens in tick spp. collected from different parts of XUAR. Furthermore, the present study did not determine the risk factors associated with screened pathogens on either animal or farm level. Subsequently, more studies are warranted in the future to avoid the risk factors correlated with TBPs in XUAR.

This study revealed the presence of *A. ovis, A. bovis, A. phagocytophilum B. motasi*-like, *B. motasi*-like Xinjiang, *T. uilenbergi*, and *T. luwenshuni* in sheep from six border counties (Qinghe, Fuhai, Jimunai, Bole, Tashikuergan, Yecheng) of XUAR, China. The current results present valuable information about ovine tick-borne infections in XUAR, which might suggest the possible emergence of tick-borne diseases in small ruminants in border regions of northwest China. Moreover, information generated from this study is expected to improve the approach for prevention and control of tick-borne diseases in China.

## Data Availability Statement

Publicly available datasets were analyzed in this study. This data can be found in the NCBI: MN946542, MN947620, MN946539, MN946540, MN946541, MN944535, and MN944557.

## Ethics Statement

The animal study was reviewed and approved by the owners of the selected farms were informed of the study and provided their written approval for sampling of their sheep. All procedures were carried out according to the ethical guidelines for the use of animal samples permitted by Obihiro University of Agriculture and Veterinary Medicine (Approval ID: 18-40). Written informed consent was obtained from the owners for the participation of their animals in this study.

## Author Contributions

YL: methodology, validation, statistical analysis of the results, writing—original draft, and writing—review & editing. EG: validation, statistical analysis the results, and writing—review & editing. QG, MR, and PM: investigation and writing—review & editing. ML, JL, and SJ: Investigation and recorded samples' data. BC: investigation, recorded samples' data, and funding acquisition. XX: conceptualization, writing—review & editing, and funding acquisition. All authors contributed to the article and approved the submitted version.

## Conflict of Interest

The authors declare that the research was conducted in the absence of any commercial or financial relationships that could be construed as a potential conflict of interest.
