# Supplementary material for: Molecular Detection and Identification of Babesia spp., Theileria spp., and Anaplasma spp. in Sheep From Border Regions, Northwestern China
Source: Front Vet Sci. 2020 Sep 16;7:630. doi: 10.3389/fvets.2020.00630 (PMC7526627; doi:10.3389/fvets.2020.00630)
Supplement: Supplementary file 1 [file Table_1.doc]

| **Pathogen** | **Target gene** | **Species-specific primers (5' → 3')** | **Fragment (bp)** | **Annealing temperature (◦C)** | **Note** | **Reference(32)** |
| --- | --- | --- | --- | --- | --- | --- |
| ***A. ovis*** | *MSP4* | TGAAGGGAGCGGGGTCATGGG  GAGTAATTGCAGCCAGGCACTCT | 347 | 62 |  | Torina et al. 2012 |
|  |  |  |  |  |  |  |
| ***A. bovis*** | 16S rRNA | TCCTGGCTCAGAACGAACGCTGGCGGCRCAGTCACTGACCCAACCTTAAATGGCTG  CTCGTAGCTTGCTATGAGAAC  TCTCCCGGACTCCAGTCTG | 1433  551 | 55  55 | 1^st^ PCR  Nested PCR | Reye et al. 2012 |
|  |  |  |  |  |  |  |
| ***A.phagocytophilum*** | *epank1* | GAGAGATGCTTATGGTAAGAC  CGTTCAGCCATCATTGTGAC | 444 | 54 |  | Walls et al. 2000 |
|  |  |  |  |  |  |  |
| ***A. marginale*** | *MSP4* | CTGAAGGGGGAGTAATGGG  GGTAATAGCTGCCAGAGATTCC | 344 | 60 |  | Torina et al. 2012 |
|  |  |  |  |  |  |  |
| ***A. capra*** | 16S rRNA | GCAAGTCGAACGGACCAAATCTGT  CCACGATTACTAGCGATTCCGACTTC | 1261 | 58 |  | Yang et al. 2016 |
|  |  |  |  |  |  |  |
| ***B. ovis*** | ssu rRNA | TGGGCAGGACCTTGGTTCTTCT  CCGCGTAGCGCCGGCTAAATA | 549 | 62 |  | Aktas et al. 2005 |
|  |  |  |  |  |  |  |
| ***B. motasi-like*** | *rap1-b* | TGCGCCTTCGAGTTGTACAAGAG  GACGGGTTGCRTAGGCTGAC | 765 | 58 | 1^st^ PCR | Q. Niu et al. 2016 |
| ***Lintan/Ningxia/Tianzhu*** |  | TGCGTGGAAGATAGAAAGTTAGCC  ATGACTGATCTCGACTCTCCATTAGCTGG | 536 | 60 | Nested PCR |  |
| ***Hebei*** |  | TCCGTGGGAGATGGAAAATCAAGC  TCGCCCGCTCCCAACTCTCCAGTACCTGA | 536 | 62 | Nested PCR |  |
| ***xinjiang*** | *rap1-a* | CGGTAGTGTGTTGATATCCTAGAG  CACAAGCGTTACATCGTAGAGCCG  AATGACCGCCCGCGAAATCAGCCACGAC  GTGGGTGGCGAAGGACAATTTGTTG | 1400  507 | 57  63 | 1^st^ PCR  Nested PCR | Q. Niu et al. 2017 |
|  |  |  |  |  |  |  |
| ***T. luwenshuni*** | 18S rRNA | GGTAGGGTATTGGCCTACTGA  TCATCCGGATAATACAAG | 389 | 57 |  | H. Yin et al. 2008 |
|  |  |  |  |  |  |  |
| ***T. uilenbergi*** | 18S rRNA | GGTAGGGTATTGGCCTACCGG  ACACTCGGAAAATGCAAGCA | 388 | 55 |  | H. Yin et al. 2008 |
| ***T. ovis*** | ssu rRNA | TCGAGACCTTCGGGT  TCCGGACATTGTAAAACAAA | 520 | 60 |  | Aktas et al. 2006 |
|  |  |  |  |  |  |  |
| ***T. orientalis*** | *MPSP* | CTTTGCCTAGGATACTTCCT  ACGGCAAGTGGTGAGAACT | 776 | 58 |  | Ota et al. 2009 |

Table S1. Primers of tick-borne pathogens

Table S2. Coinfections of *Anaplasma* spp., *Babesia* spp. and *Theileria* spp.

| Type of Pathogen | No. Infected/(%) | Sampling area, No. Infected/% (95% CI) | | | | | |
| --- | --- | --- | --- | --- | --- | --- | --- |
|  |  | TS | BL | FH | JM | YC | QH |
| *A.ovis*+*A.bovis* | 40/12.4 (0.09-0.16) | - | 6/17.6 (0.04-0.31) | 10/34.5 (0.16-0.53) | - | 11/13.6 (0.06-0.21) | 13/16.0 (0.08-0.24) |
| *A.ovis*+*A.ph* | 22/6.8 (0.04-0.09) | - | 6/17.6 (0.04-0.31) | 1/3.4 (-0.04-0.11) | 1/4.5 (-0.05-0.14) | 14/17.3 (0.09-0.26) | - |
| *A.ovis*+*B.m* | 55/17.0 (0.13-0.21) | - | 25/73.5 (0.58-0.89) | 8/27.6 (0.10-0.45) | 4/18.2 (0-0.36) | - | 18/22.2 (0.13-0.31) |
| *A.ovis*+*B.m XJ* | 19/5.9 (0.03-0.08) | - | 12/35.3 (0.18-0.52) | 3/10.3 (-0.1-0.22) | 1/4.5 (-0.05-0.14) | - | 3/3.7 () |
| *A.ovis*+*T.lu* | 14/4.3 (0.02-0.07) | - | 3/8.8 (-0.01-0.19) | 6/20.7 (0.05-0.36) | - | 2/2.5 (0-0.06) | 3/3.7 (0-0.08) |
| *A.ovis*+*T.u* | 38/11.8 (0.08-0.15) | - | 16/47.1 (0.29-0.65) | 1/3.4 (-0.04-0.11) | - | 7/8.6 (0.02-0.15) | 14/17.3 (0.09-0.26) |
| *A.bovis*+*B.m* | 12/3.7 (0.02-0.06) | - | 5/14.7 (0.02-0.27) | 4/13.8 (0-0.27) | - | - | 3/3.7 (0-0.08) |
| *A.bovis*+*B.m XJ* | 7/2.2 (0.01-0.04) | - | 4/11.8 (0-0.23) | 2/6.8 (-0.03-0.17) | - | - | 1/1.2 (0-0.04) |
| *A.bovis+T.u* | 11/3.4 (0.01-0.05) | - | 3/8.8 (-0.01-0.19) | 4/13.8 (0-0.27) | - | 2/2.5 (0-0.06) | 2/2.5 (0-0.06) |
| *A*.*ph*+*B.m* | 8/2.5 (0.01-0.04) | - | 7/20.6 (0.06-0.34) | 1/3.4 (-0.04-0.11) | - | - | - |
| *A.ph+T.u* | 9/2.8 (0.01-0.05) | - | 6/17.6 (0.04-0.31) | 1/3.4 (-0.04-0.11) | - | 2/2.5 (0-0.06) | - |
| *T.u+B.m* | 17/5.3 (0.03-0.08) | - | 13/38.2 (0.21-0.55) | 2/6.8 (-0.03-0.17) | - | - | 2/2.5 (0-0.06) |
| *A.ovis*+*B.m+T.u* | 16/5.0 (0.03-0.07) | - | 14/41.2 (0.23-0.59) | 2/6.8 (-0.03-0.17) | - | - | 2/2.5 (0-0.06) |
| *A.ovis*+*A.bovis+B.m* | 11/3.4 (0.01-0.05) | - | 4/11.8 (0-0.23) | 5/17.2 (0.03-0.32) | - | - | 2/2.5 (0-0.06) |
| *A.ovis*+*A.bovis+T.u* | 8/2.5 (0.01-0.04) | - | 2/5.9 (-0.02-0.14) | 3/10.3 (-0.01-0.22) | - | 1/1.2 (0-0.04) | 2/2.5 (0-0.06) |
| *A.bovis+B.m+T.u* | 5/1.5 (0-0.03) | - | 3/8.8 (-0.01-0.19) | 2/6.9 (-0.03-0.17) | - | - | - |
| *A.ovis*+*A.bovis+A.ph* | 3/0.9 (0-0.02) | - | 1/2.9 () | - | - | 2/2.5 (0-0.06) | - |

“-” mean not detected; Tashikurgan (TS, n=76), Bole (BL, n=34), Fuhai (FH, n=29), Jimunai (JM, n=22),Yecheng (YC, n=81) and Qinghe (QH, n=81).

Table S3. The tick-borne pathogens gene accession number

| Species | Gene | Accession number |
| --- | --- | --- |
| *A. ovis* | *MSP4* | MN946542 |
| *A. bovis* | 16S rRNA | MN947620 |
| *A. phagocytophilum* | *epank1* | MN946539 |
| *B. motasi-like* | *rap1-b* | MN946540 |
| *B. motasi-like Xinjiang* | *rap1-a* | MN946541 |
| *T. luwenshuni* | 18S rRNA | MN944535 |
| *T. uilenbergi* | 18S rRNA | MN944557 |
